# New York Odontological Society

**Published:** 1895-07

**Authors:** 

**Affiliations:** New York Odontological Society


### Reports of Society Meetings.

### NEW YORK ODONTOLOGICAL SOCIETY.

A REGULAR meeting of the New York Odontological Society was held on Tuesday evening February 19, 1895, at the New York Academy of Medicine, No. 17 West Forty-third Street, New York, with the President, Dr. Northrop, in the chair.

The minutes of the previous meeting were read by the secretary and approved.

*The President.*—I take great pleasure in presenting to you to-night some models of a mouth, from J. G. Briggotte, of Paris. These casts were described by Dr. Briggotte in a paper read before this Society March 9, 1886, an account of which can be found on page 365 of vol. xxviii. of the *Dental Cosmos.* They are put up in composition, and show the articulation (which is very much out of place), and what was done to provide the mouth with a perfect denture. It seems to me he has displayed a great deal of skill and discretion, and the models are worthy of thought and examination.

A motion to receive the same with thanks was carried.

*Dr. John H. Meyer.*—There have been many contrivances brought before the dental profession to enable the practitioner to make his own porcelain crowns, porcelain inlays, porcelain tips, bridge-work, the making of blocks, or the building up of broken teeth, which would be more generally adopted if the process of baking were better understood. I have been so frequently consulted regarding this that I have decided to offer my services to teach the art of baking porcelain to any member of this or a sister society free of charge, and will set aside each Saturday afternoon from two until four o'clock, during the months of March, April, and May for that purpose.

The paper of the evening, "The Application of some Principles of Law to the Practice of Dentistry," was then read by Mr. W. A. Purrington, of New York, who prefaced the reading with the following remarks.

*Mr. Purrington.*—I find myself very much in the position of a greedy boy with a very red apple, who has bitten off a great deal more than he can comfortably chew. I gave your secretary a synopsis of this paper in an oblivious moment, not realizing altogether its scope, which is so wide that it would be absolutely impossible for me to enlarge upon the various topics without boring you to death and keeping you here a weary age. Consequently I can only give to you in detail that which seems to be of most importance, and touch upon the other subjects with what lightness I may; because, if I knew the entire law (as I do not), I should still be unable to tell it to you; and if I should tell you all the law on the subject, you would go away very little wiser than you came; so it is better for me to confine my paper to those topics which seem peculiarly interesting to your profession.

I want to say one word as to the progress of dentistry towards the professional status. We know perfectly well that the practice of medicine and surgery was formerly carried on by the priests. The healing of the body and the healing of the soul was in the same hands; but the healing of the body proved more fascinating to many than the healing of the soul, and monks and priests would absent themselves from the convents to look after the sick. They did not say so many *Aves* and they skipped many *Paters;* but they did a great deal of good. So the Council of Tours forbade that the priests should perform any operations; thus surgical operations and later the practice of physic passed into lay hands. You may see very markedly the result even to-day in the older communities; in England, for instance, where a physician has a very different standing from a surgeon or an apothecary. While physic was still practised by the clergy, surgery fell into the hands of the smith and the barber, whose pole, with a bandage, is still displayed in remembrance of the twofold trade. The English surgeon is not called doctor. He is simply mister. His social rank is less than that of physicians, some of whom have been knighted, although I do not recall any physician who has gone beyond knighthood. We know that all this is ridiculous, we see its folly, the asininity of still regarding men who devote themselves to the healing of the body somewhat from the point of view taken in a time when medical men were the constant object of ridicule. But the force of custom is powerful and persistent. In new communities it is less so.

But neither custom nor laws can say the last word in determining our lives.

We make ourselves. The chief use of any law is the schoolmaster use. I would not give the snap of my finger for our license law if its sole effect were that visited upon the individual punished for its violation. You find him transgressing again and again. He pays his fine each time. But when he is convicted and the newspapers say that he is convicted, a large number of men fear to follow his example, and a larger number grow to consider his behavior as low and disreputable. There is the influence of the law.

The initial chapter of a recent publication, *Code du Chirurgien Dentiste*, par MM. Roger and Godon, written to show the position of the dentist under the French law, points out how analogous that position has been in the early part of this century to that of the physician in the time of Molière, when the man of drugs was a fair butt for the shafts of every wit. There was ground for this ridicule in the ignorance and pretentiousness of physicians. There was the same ground as to dentists. The advertising by stuffed crocodiles and alembics of the one, the displays of grinning teeth by the other were fair game for the wags. As physician or dentist came to be more skilful, learned, and modest, they won and received higher recognition. And in our day of diffused scientific knowledge the surgeon bids fair to outstrip the physician. If a lawyer misconducts his case in court, every lawyer there may know it. And so do the surgeon and the dentist leave their open trail for their fellows to detect, while the physician's work still continues to be largely in the field of conjecture where error is hard to trace. Some of you will remember the famous case of Professor Webster, convicted of murdering Mr. Parkman. The latter dunned the former persistently, and Webster, probably in a fit of passion and, perhaps, without criminal intent, killed him. He then, to conceal the crime, cut the body to pieces and burned it piecemeal. He was detected at the work, a tooth was found and submitted to a dentist who, recognizing his work, exclaimed "poor Parkman," and Webster's fate was sealed. It is because of the trail that the surgeon and dentist leave by mechanical operations that malpractice suits are more often brought against them than against physicians, whose faults the earth so often covers. Medical men must be differentiated in a way; but the differentiation is not to be made in the cradle. A medical student does not or should not begin his specialization in the class-room. He gets his determining bent as his knowledge grows. I am but a layman, and yet can see some reason for the tremendous and perhaps excessive specialization of our time; but it does seem to me that the best specialization is that of a man who starts at the foundation, and is first a physician and then a specialist, as his riper knowledge or the accidents of practice may determine. The broader the basis of knowledge the greater your mental and scientific position in every way; you are of the family of medical men, and your early education should be with your fellows; that being so, whatever specialty you may follow you will still be in the fold.

I can scarcely quit this good company and this topic without suggesting that, if the enactment and enforcement of dental laws has benefited your profession and the public in this State, you owe a debt, more than many of you realize, to those of your number—to one notably—who, in a spirit of unselfish devotion, have achieved such results as we have, sparing neither time nor money, and paying liberally the debt that every man is said to owe to his profession. I have been in position to see that faithful work, and I am sure I need not mention a name to call up in your minds to whom your appreciation is so largely due.

(For Mr. Purrington's paper, see page 403.)

DISCUSSION.

*Dr. Carr.*—I wish to ask Mr. Purrington a question. If a patient requested that a certain tooth, which he designated, should be extracted, and, after extraction, the tooth was found to be sound, could the practitioner legally be held responsible?

*Mr. Purrington.*—The only answer I can make is, that it depends somewhat upon the doctrine of "contributory negligence." If your mistake were due to the patient's fault you would be excusable.

If a man came to your office with an aching tooth and said, "Doctor, extract my tooth," and you—especially if the man were under the influence of anæsthesia—should extract a sound tooth, the man might, perhaps, recover damages for your negligence; but, on the other hand, if the man wanted a tooth extracted, all the other teeth being in a similar condition, you have a right to extract whatever tooth he should point out; but such questions of fact can only be solved by the jury. You would be negligent if a man had two teeth side by side, one absolutely sound and one absolutely bad, if you should extract the sound tooth because he happened to point it out, leaving the bad one in the mouth. That would be scarcely contributory negligence on his part, I should say, for under such circumstances the patient may easily touch the wrong tooth.

*Dr. Carr.*—Although the student clause is clearly defined, yet many claim that the student is not amenable to the law if he operates in the office with a dentist. Will Mr. Purrington define the student clause?

*Mr. Purrington.*—This is asking me to pass on a statutory law When MacCauley made his Indian code, he thought lawyers were fools to find so much difficulty in making and construing statutes. But when a case was submitted to him he admitted that he knew what it meant when he wrote the code, but did not know what it meant when the lawyers presented the two sides of the question. It seems to me that the student law is so clear that there can be no doubt about it. The student is exempted who assists his preceptor in clinical operations. That is a full exemption. There is no reason in the world why I cannot assist you if I am in your office. You are trained and capable, and I being there, and you wanting some assistance, whatever it may be,—there is no reason in common sense why I cannot help you. The most instructive and entertaining after-dinner speech I ever heard was made at a dinner of the medical alumni of the University by one who is now dead. He spoke of the happy life of a rural physician, and of an operation performed by him now very common,—then very rare. He said that he had extracted a tumor from a woman who weighed one hundred and sixty pounds when she came in, and ninety pounds when she went out, and his only assistants were the laymen he could call in to help him.

There is no reason why I cannot help any suffering person in the absence of a professional man. This statute expressly provides that your student, with you for purposes of clinical instruction, may help you; but it does not mean that you can turn your work over to your student. He may help you; he cannot act for you. You do the work; he stands by. If there were one thing wherein your profession had the advantage over the medical profession in the present system of training, I should say it were in that one particular thing that your student has opportunities for clinical instruction that do not exist in the medical profession, except for the favored few who walk to the hospitals.

The law intends that when you perform an operation, and have a student in your office, you have a right to ask him to help you; but it must be under your immediate eye. You do the work and he gives such assistance as you may require, and therein he gets his knowledge.

*Dr. Crawford.*—I can say with much pleasure that I am glad to meet the essayist. I have been very much entertained this evening. If I were going to write a book on the subject of medical or dental jurisprudence, I would like to have the essay of the evening as a preface. I think it would be a good thing if this Society would request all the dental journals in this country to publish this paper. I would like to take it down to my office and post it on my walls.

I was particularly pleased with the definition the gentleman gave of what dental surgery is and where it belongs. That is the kind of doctrine that I like to endorse. Any other that has ever been given on that subject has had a tendency to impair the interest of the so-called dental profession. It is one of the most important questions that has ever been agitated in our specialty. At the time it gave me a great deal of trouble and concern, and I want, in his presence to-night, to earnestly thank the learned gentleman for the truth in reference to this matter. The moral force of everything else that has been said on that subject has had rather a tendency to injure and impair the welfare of this department of medicine. I want to say for the gentleman's encouragement that of all the departments of medicine and surgery, the dental surgeon needs a higher, a broader, and a better fundamental education than any other specialty in surgery. The results of our modern civilization have made it so that the American child needs a dental surgeon to superintend the eruption, and particularly the displacement or shedding of the first set of teeth, as he needs the doctor of divinity to baptize him, or the obstetrician to preside over the parturient chamber when he first sees the light of day. Upon that hypothesis I claim that it more nearly touches the entire human family than any other branch of surgery. I thank you for the definition of what dental surgery is, and for placing it where it belongs.

*The President.*—We have with us to-night Mr. John Sabine Smith, who is president of the Society of Medical Jurisprudence, and we shall all be pleased to hear from him.

*Mr. John Sabine Smith.*—I came here to listen and to learn. I feel that I have learned a great deal this evening. This is a new branch of jurisprudence to me, and one to which I have never given any special thought; but I think the subject has been so exhaustively treated in the paper that nothing can be added to it. Only one suggestion presents itself to me, and that is, the question whether dentistry is to be regarded as a distinct profession, or whether it is a branch of medicine. What little observation I have had on the subject has led me to think that it is a question to be determined by the profession itself. There are no better judges of the subject where the line is to be drawn than the dentists themselves. At least, they are able to determine that question as they stand before the public,—to determine whether it is to be a profession or not. If they place it upon a high plane and keep it there, with all the dignity and propriety and seclusion which applies to medicine, law, and theology, I think we will find that it will be conceded to be fully equal to rest upon the same plane as the others, and to be entitled to all the privileges and respect which other professions receive. The illustration of that comes from the case spoken of by the essayist, which occurred in Missouri, I believe, in which three judges determined that it was a profession by itself, and when seven judges sat together, four of them reversed the decision, and declared that it was not, and that it might be classed with chiropodists and other kindred grades of skill. I am impressed with the idea that the four judges who had reversed the case had been in the habit of employing cheap advertising dentists. They had a low opinion of the profession. They regarded the dentist's instruments as tools. They classed a dentist with a jeweller, for instance. Probably they had work done cheap. Perhaps one of them had some false teeth made for a small price, and in order to do it, had had his own teeth extracted at a discount, and he made up his mind that dentistry was a good trade. He never thought of it being anything else. But on the other bench, where there were three judges, one of them had a great respect for his mouth as much as any other part of his body. He had consulted skilful dentists, and he came to regard the dentist as he did his physician. I think this question will be ultimately determined as a matter of history, so to say, as among the affairs of the world. It will ultimately be written down as a profession entitled to all the dignity and respect of the other learned professions, when it becomes universally such among dentists themselves.

*Professor Abbott.*—All this discussion is extremely interesting, and much of it very instructive. Still, the whole drift seems to be towards the idea that dentistry as a specialty of medicine stands on the same level, or has a right to be considered on the same level, as the older or the mother profession of general medicine. You must remember that dentistry as such is comparatively young in this country. How did practical dentistry commence? By jewellers, watch-makers, tinkers, and barbers. Even to-day some barbers are practising it. It takes time for any profession or specialty of a profession to grow. It is taught as a specialty in our colleges; and in our teaching we go very far towards qualifying the dental student for general practice. We teach anatomy, physiology, pathology, and chemistry as thoroughly as any medical schools. All medicines used in treating diseases of the buccal cavity, whether directly or indirectly, are carefully considered. Instead of going over the ground of the general practitioner, in general practice of medicine and surgery, obstetrics and the diseases of women and children, oral and dental surgery, and dental prosthesis are taught. When our young men leave college and come to me for advice in reference to their further studies, I advise them all, if possible, to keep on until they get their degree of M.D. They have simply begun their studies, have merely education enough to practise, perhaps successfully; but they need a broad foundation, and the way to get it is to enter some good medical college and secure a degree. I see before me to-night more than one man who holds his degree of M.D. under that advice. There are some things in reference to professional education that I think should be looked into. I was very much pleased, indeed, with the definition given by the essayist in reference to the student's position. Many students have come to me with what they understood was a construction of the law in the State of New York. This construction was that they could go into an office and practise as much as they pleased, as long as the dentist himself was somewhere in the house. My only comment at such times is, "It seems to me you should work as an assistant in an office, and if you do anything more than assist your preceptor you take risks." "Never do anything where there would be a chance of prosecution for violating the law;" students do, I believe, work outside in offices "over the chair." I am satisfied that it is a very common practice with many men. In Brooklyn, Jersey City, and New York you can find many of them who spend three or four hours every day in such practice. These men are certainly violating the law of this State and of New Jersey. In New Jersey every man is required to pass a good solid examination, as severe as any college faculty would give him before he is allowed to practise in that State. It is the kind of examination that I hope to see conducted in this State. Why? Simply because we want to know who is competent to practise dentistry before he is allowed to do so. We do not want men to come here and merely because they have a diploma be allowed to go to the county clerk's office and register. Many men holding diplomas are not competent, and should never touch a patient's mouth. They should not be allowed to practise until it is determined by a competent Board of Examiners in the State of New York that they have an educational right to do so.

*Dr. Rhein.*—I would like to ask the essayist a question in reference to students working in offices. If a student has the privilege in a college to clinically work over patients for which a fee of some kind is required, would a strict interpretation of the Code say that a student could not further practise gratuitously outside of the college?

*Dr. Carr.*—In colleges there are demonstrators who are legally qualified to practise dentistry and who go from chair to chair giving instructions to students.

*Mr. Purrington.*—There is no law that allows a man to do anything in a college that he cannot do outside. I think the idea that you allude to is a development. It is a very false idea, having its origin perhaps in the fact that under our medical statute there has been an exemption of men on the hospital staffs. There is nothing in the law that would allow a student inside of a college to practise gratuitously, or for a fee. If he does so, he may thank his environment that he is not caught. If he goes outside where there is not that environment, he is liable to be caught, because almost any day a person employed by the dental society might come in and catch him, and he would be punished. I would infer from your question that there must be a certain amount of violation of the dental laws going on inside of the college walls.

*Dr. Rhein.*—Why is it that so much stress is always laid on the point (when bringing proceedings against illegal practitioners), that they have received a fee for these services, if it makes no difference that the work has been done gratuitously or for a fee?

*Mr. Purrington.*—There are two reasons for it. In the first place, the statute as originally drawn, distinctly provided that the work should be for a fee or reward. The other reason is one alluded to in my paper, that practice implies a customary act. In a case that you could suppose, whether certain acts constitute practice in dentistry must, in the last resort, be a case for the jury. You might tell me of a hundred cases, and I could not really say how in those cases a jury would pass upon the facts. I may practise dentistry for a fee or reward; or I may, in a case of great emergency, finding a man suffering with toothache, assist him. The latter act, of course, does not mean that I am practising dentistry. I simply suggest to a suffering fellow something that will help him. Whether defendant has practised is the question in every case for the police, the judge, or the jury. But there is no intention on the part of the law to allow unqualified men to practise. It is allowed that students may assist preceptors in their work, to the end that the students may get instruction therefrom. They are there to see how those things are done. I do not know enough about the detail of the college work to say exactly whether or not the law is ever violated there.

*Dr. Abbott.*—The answer to this question is not altogether satisfactory, for the reason that dentistry is a peculiar calling. No man can be a dentist until he handles the patient himself. No man can learn dental surgery except by actual practice. He may look at his perceptor operate for years, and still be ignorant of the way of managing the patient himself. The student who looks into the patient's mouth and sees the operation done by the practitioner will eventually get a patient, and he will work upon him. No matter if it is contrary to law, he must learn in some way or other; he can pass no examination before any college faculty or any board of examiners without that practical knowledge. How is he to obtain that knowledge if he is not allowed to practise? In our colleges that is a special department. We have one room with from seventy-five to one hundred chairs in it. Sometimes that room is filled with patients and operators,—young men who are learning practical dentistry. They are practising under the instruction of men who go from one to another and tell them how to do the work, how to avoid making mistakes, etc. If every student in our colleges is violating the law by practising over patients, it is a remarkable state of affairs, and not what, in my judgment, was intended by the framers of our laws. I hardly think a jury would convict a man who did it. We have any quantity of men in this city (and some of them I see before me now) who have been at the college and know just what is required. They learned their first practical ideas of dentistry there by working over the patients themselves. The work is always done under instructors, so that students can hardly make mistakes. Of course, the greatest care is taken in the selection of the men who instruct them. A careful study of this subject, it seems to me, will convince any man that such practical instruction is of the greatest importance from a public health stand-point.

*Mr. Purrington.*—It seems to me as if this is based on a hypothetical question on one side, followed by an entirely different hypothetical question on the other. If a man came into a dental college and paid fees, and were practised on, how would that be different from practice carried on outside? Dr. Abbott puts the case differently, and says a person is practised on under the eye of the instructor. The fundamental point that Dr. Abbott raises is so true that I do not see how we will ever get away from it. I have always felt uncomfortable at the thought that all medical students have to get their experience at the expense of persons who would recover in spite of them, or would die in spite of their best efforts. It is said that a man cannot practise medicine unless he has certain qualifications. All the law in the world cannot give him those qualifications. You cannot make a silk purse out of a sow's ear. Nothing is more appalling to a young lawyer, I suppose, than his first case. He comes out of a law school; he has never practised law. A man gives him a case, and the young lawyer is almost sick. It does not follow from that, that the law will allow that law student to practise before he is admitted to the bar. The curse of the New York Legislature is that it tries to provide for every conceivable infinitesimal point that the wit of man can imagine. You cannot do that, and the more you try the more you handicap men in their ordinary functions of life. The application of the law to what happens inside the college, and what happens outside ot it, can only be made as cases arise. You cannot go before a court and say, "We have come here with a sophomoric or academic question, and we want it decided." The court would say, "Come back w h en question of damages is to be determined." As a general rule, the statute says you cannot practise dentistry, you cannot set yourself up as a dentist unless you are qualified; but the law does not say to me that I cannot tell my suffering friend to put a hot raisin against the side of his tooth to relieve his suffering, or make any other suggestion equally good or bad, or even help a dentist in a case of emergency. The statute aims to prevent a practice, not to punish a single act performer, with no intent of repetition or to violate the law.

A vote of thanks was offered to Mr. Purrington and those gentlemen who took part in the discussion.

Adjourned.

John I. Hart, D.D.S.
*Editor New York Odontological Society.*

